# The role of high-resolution cartilage thickness distribution for contact mechanics predictions in the tibiofemoral joint

**DOI:** 10.1177/09544119241307793

**Published:** 2025-01-09

**Authors:** Robert J Cooper, Gavin A Day, Vithanage N Wijayathunga, Jiacheng Yao, Marlène Mengoni, Ruth K Wilcox, Alison C Jones

**Affiliations:** Institute of Medical and Biological Engineering, University of Leeds, Leeds, UK

**Keywords:** Finite element modelling, tibiofemoral joint, subject-specific modelling, contact pressure distribution, computational model validation

## Abstract

Subject-specific finite element models of knee joint contact mechanics are used in assessment of interventions and disease states. Cartilage thickness distribution is one factor influencing the distribution of pressure. Precision of cartilage geometry capture varies between imaging protocols. This work evaluated the cartilage thickness distribution precision needed for contact mechanics prediction in models of the tibiofemoral joint by comparing model outputs to experimental measurements for three cadaveric specimens. Models with location-specific cartilage thickness were compared to those with a uniform thickness, for a fixed relative orientation of the femur and tibia and with tibial freedom of movement. Under constrained conditions, the advantage of including location-specific cartilage thickness was clear. Models with location-specific thickness predicted the proportion of force through each condyle with an average error of 5% (compared to 27% with uniform thickness) and predicted the experimental contact area with an error of 21 mm^2^ (compared to 98 mm^2^ with uniform thickness). With tibial freedom, the advantage of location-specific cartilage thickness not clear. The attempt to allow three degrees of relative freedom at the tibiofemoral joint resulted in a high degree of experimental and computational uncertainty. It is therefore recommended that researchers avoid this level of freedom. This work provides some evidence that highly constrained conditions make tibiofemoral contact mechanics predictions more sensitive to cartilage thickness and should perhaps be avoided in studies where the means to generate subject-specific cartilage thickness are not available.

## Graphical abstract

**Graphical abstract:**
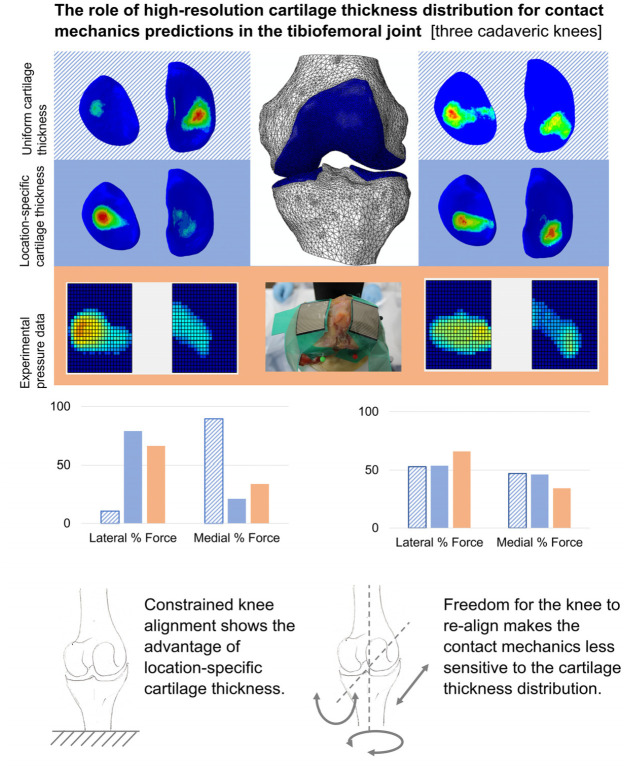

## Introduction

Finite element models of the knee joint are now a well-established research tool for the assessment of differences in contact mechanics with knee injury, such as meniscal tears, and after interventions, such as partial or full meniscectomy.^1,2^ Current state-of-the-art image-based modelling techniques can be used to build large suites of models. There is for instance increasing interest in developing virtual trials for clinical products where models represent variance in the patient population, either by using image-based models^3^ or by using statistical shape and appearance models to generate synthetic specimens.^4^

Cartilage thickness is a key indicator of osteoarthritis progression^5^ and is one of the factors expected to influence the distribution of pressure within the joint, often used as a feature of interest in finite element models of the knee joint. Cartilage thickness varies across the joint^6^ and the precision with which that variation can be captured depends on the resolution and contrast of the three-dimensional imaging modality used. Many studies directly segment the variable cartilage thickness profiles from 3D images, which requires specific magnetic resonance (MR) sequences^7^ or computed tomography (CT) imaging with the surrounding tissue removed,^8^ or a combination of these two imaging modalities to combine their advantages. This shape capture is not always possible when imaging in vivo or at multiple time points and the specialist imaging and segmentation time adds restrictions to the number of knees which can be analysed. Alternative approaches include scaling a template geometry to the dimensions of the target knee^9^ and deriving the cartilage thickness from the bone geometry in an automated manner. The simplest way to do the latter is to assume a uniform thickness and allow the cartilage articular surface to match the shape of the subchondral bone.^10^ Some work has explored the prediction of cartilage thickness profiles from the underlying bone shape,^11^ which offers an attractive solution, but requires a statistical shape model based on a sufficiently broad population and representation of cartilage wear or damage would be challenging.

There is currently little quantitative evidence available to support the use of subject-specific cartilage thickness distributions in predicting tibiofemoral contact mechanics. Therefore, the aim of this work was to quantify the effect of using uniform thickness cartilage versus high-resolution non-uniform specimen-specific thickness on contact mechanics predictions, across three human knees. To that end, models with location-specific cartilage layers were compared to those with a uniform thickness in terms of their ability to replicate experimentally measured contact mechanics.

The intention was to compare finite element contact mechanics measurements under two constraint conditions; one where the relative movement of the two bones was fully constrained and one where the tibia had extensive freedom to re-align under load, representing two extremes at either side of most in vivo constraints. The latter condition proved challenging, and those challenges are described for the benefit of future research.

## Methods

### Overview of approach

**Figure 1.**
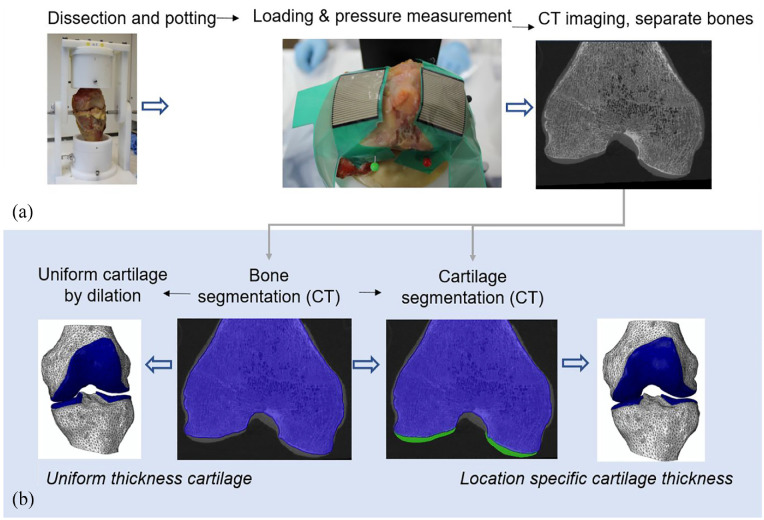
An overview of (a) the experimental protocol and (b) the development steps for the two cartilage representations in the computational models.

Image-based finite element models were generated of three human cadaveric tibiofemoral knee specimens that were mechanically tested in the laboratory using the workflow summarised in Figure 1. Briefly, each joint was dissected to just the tibia and femoral bone and cartilage and tested experimentally, under two levels of tibial constraint. Finite element models of the specimens were generated from the image data with the cartilage thickness either derived directly from CT images (‘location specific’ case) or assigned a uniform value (‘uniform thickness’ case). Detailed protocols are presented for the experimental work (Section ‘Experimental methods and specimen imaging’) and computational work (Section ‘Computational modelling methods’) and are also available in the University of Leeds Data Repository.^12^

### Experimental methods and specimen imaging

Following ethical approval (East Midlands – Leicester South Research Ethics Committee (18/EM/0224)), three fresh-frozen human cadaveric knees were obtained and stored at −40°C. Tissue was obtained from male donors aged 57 (right knee), 61 (left knee) and 81 (left knee) (respectively with BMI of 38.1, 18.0 and 23.7). Each knee was defrosted in the fridge for approximately 48 h before MR imaging was performed without any tissue manipulation to assess if tissue quality was suitable for experimental testing, after which they were returned to −40°C storage. Each knee was transferred to a 4ºC fridge for defrosting, 48 h prior to the day of dissection and cement potting. During dissection the patella was removed, along with the majority of soft tissues, retaining the articular cartilage, the menisci and the posterior capsular tissues, to prevent over extension of the joint during manipulations and potting. Established methods were used to locate an axis of femoral flexion, recorded by drilling a small hole on either side of the femoral condyles.^13–15^ The femur and tibia were then cemented in custom-built pots using polymethylmethacrylate (PMMA; WHW Plastics, UK), using a rig the same size as the testing set up and using the previously drilled holes to ensure femoral alignment. The tibiofemoral joint was held at zero flexion to provide a seated alignment of the tibia relative to the femur. The menisci and posterior capsular soft tissue were resected following potting. Potted specimens were stored overnight in the fridge (4ºC) and removed from the fridge to reach room temperature (∼3 h) before testing, during which mounting and setting up of the sample on the test rig was also carried out.

Thin film pressure sensors (Tekscan Pressure Mapping Sensor Model 4000, Tekscan Inc., Boston, MA, USA) were inserted in each condyle and fixed using pins anteriorly and posteriorly. Prior to testing, the pressure sensors were conditioned and calibrated using the Tekscan software I-Scan against a pre-calibrated load cell (Instron, UK) rated to 5 kN. A new pressure sensor was used for each knee specimen to minimise error resulting from deformation of the sensors when positioned on the uneven knee joint surfaces.

**Figure 2.**
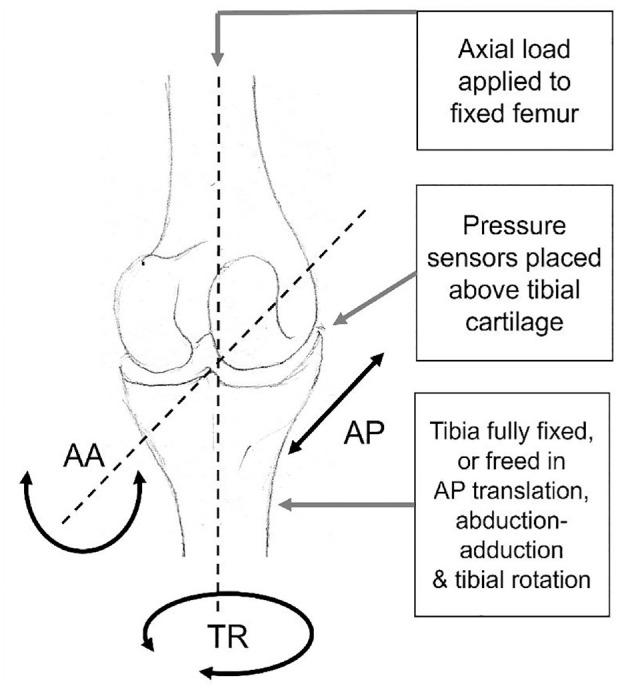
Example of the set up for the experimental tests showing a human tibiofemoral joint schematic, with the location of pressure sensors used to measure contact pressures. The three degrees of freedom which can be released on the tibial side are adduction–abduction rotation (AA), tibial rotation (TR) and anterior–posterior translation (AP).

Each potted knee was placed in a custom rig,^13^ where the femur was constrained in all degrees of freedom except for superior-inferior translation. Two cases of tibial constraint were tested: one ‘fully constrained’, with all degrees of freedom on both bones constrained, except the superior–inferior direction on the femur, and one ‘partially freed’, where the tibia was allowed to move in three degrees of freedom, namely anterior–posterior translation, internal–external rotation and abduction–adduction (Figure 2). Each knee was axially loaded in a material testing machine (Instron 3365 with a 5 kN load cell, Instron, UK). An axial compression was slowly increased (1 mm/min) up to 500 N and then held at 500 N for 60 s when contact pressure measurements were taken.^13,16^ For the partially freed case on Knee 2, the full 500 N load could not be achieved since a large amount of anterior displacement meant that the experiments had to be terminated to avoid dislocation. Results at the maximum possible load of 80 N were therefore recorded for Knee 2.

After testing, each bone was imaged separately using high-resolution peripheral quantitative computed tomography (XtremeCT, Scanco Medical AG, Switzerland) at an isotropic voxel size of 82 µm. The joint separation allowed for CT images to be used to clearly identify cartilage layers on each bone.

### Computational modelling methods

Femoral and tibial CT images were aligned and segmented using Simpleware ScanIP 2019.09 (Synopsys, Mountain View, CA, USA). The relative alignment of the tibia and femur bones was established from features visible on the CT images, including the experimental femoral flexion axis (drill holes) and the axial plane for each bone (cement base). Femoral and tibial bones and cartilage layers for each knee were segmented using thresholding followed by morphological operations and Gaussian smoothing for noise removal, creating ‘location specific’ cartilage models. Alongside these, ‘uniform thickness’ cartilage models were created with the cartilage generated by dilating out the bone masks in the regions where cartilage was visible. The uniform cartilage thickness value was different for the femur and tibia of each knee and based on the observed averages on CT images.

Finite element models of each case were developed in Abaqus 2017 (Dassault Systèmes, Vélizy-Villacoublay, France) by importing meshed geometries from ScanIP. All the FE models developed for this study were static analyses, with geometric non-linearity and unsymmetric matrix storage.

All tissue masks were meshed with quadratic tetrahedral elements.^17^ Mesh sensitivity testing conducted in a previous porcine study^13^ showed low sensitivity of peak pressure to element size. Doubling the number of elements, equivalent to a 20% reduction in element edge length, resulted in a 1% change in peak contact pressure, a 3% change in mean pressure, and 2% change in contact area. In this human tissue work, the average element edge lengths in the collagenous tissues were consistently smaller than in the porcine work (1.1–1.2 mm, compared to 1.3 mm for porcine). The cartilage thickness was similar to the porcine models when a uniform thickness was used and thicker in the load bearing areas of the cases with location-specific thickness. Overall, the models contained approximately 180,000 elements, of which 110,000 elements were for the cartilaginous soft tissues. The mesh density was considered a good compromise between precision and computational cost. The mean runtimes for cases with a fully constrained tibia and partially freed tibia were 3 h (3.3 GHz CPU, 16 GB ram, 4 CPUs) and 9 h (3.2 GHz CPU, 128 GB ram, 16 CPUs) respectively.

The material properties were consistent between all three knees, to avoid introducing a confounding factor to this study (investigating the effect of shape). Bones were modelled as homogeneous, linearly elastic materials^18,19^ representing much stiffer behaviour than the cartilage (*E* = 15 GPa, ν = 0.3). The femoral and tibial cartilage layers were modelled as Neo-Hookean hyperelastic materials^20^ (*C*_10_ = 1.0274 MPa, *K* = 25 MPa). In unconfined compression and indentation testing Young’s Modulus values have been found to be <1 MPa for osteoarthritic samples and up to 18 MPa for healthy tissue.^21^ The parameters selected here represent an approximate linear modulus of 6 MPa. Frictional hard contact enforced using the penalty method was defined between femoral and tibial cartilages (μ = 0.1), representing the lower end of friction coefficient values recorded for the human femoral condyle.^22^

The experimental constraint cases and loads were replicated for each knee creating a total of 12 models (three specimens each with two cartilage definitions and two types of constraints). Two reference points were placed at the same location on the femoral axis of rotation. One reference point was linked through kinematic coupling to the nodes on the proximal surface of the femur and used to apply the femoral constraints and a concentrated compressive force. The other reference point was coupled to the nodes on the distal surface of the tibia and used to apply the tibial constraints. In the ‘partially freed’ case, the solution would not converge consistently if all three tibial degrees of freedom were unconstrained simultaneously. Therefore, each tibial degree of freedom was applied in a separate analysis step, while all other degrees of freedom were constrained, and the resulting displacements and rotations were inherited and modified from step to step. Four loading steps were used, freeing in order: adduction–abduction; anterior–posterior displacement; internal–external rotation; and adduction–abduction once again. Adduction–abduction was prioritised as it would have a direct influence on the balance of force between the two condyles and generated the highest reaction moment in the fixed cases (see Supplementary Material). Metrics were taken from the final step.

### Measurements and analysis

The local cartilage thickness across the femoral and tibial condyles was measured using a sphere expansion algorithm (Simpleware, ScanIP) and the thickness distribution was visualised for context.

The computational model predictions of pressure distribution (qualitative), balance of force between the two condyles, and contact area on the tibial cartilage, were compared to the corresponding experimental data to assess the importance of the location-specific cartilage thickness depending on the experimental constraints represented in the models.

The number of contact patches and their shape were qualitatively compared between the experimental and computational images showing the contact area through pressure distribution maps. While the sensors covered the majority of the tibial cartilage (Figure 1(a)) it was not possible to perfectly match the location of contact patches between the experimental and computational cases, therefore only large changes in patch location were noted.

The computational contact area on each condyle was computed as the sum of the nodal contact area for all nodes of the corresponding tibial condyle. The experimental contact area data was measured from the area of all ‘sensels’ (load sensing unit cell areas) with non-zero pressure. For the sensels on the edge of the contact region, it was unclear how much of each sensel was truly within the contact area. Therefore, the contact area was calculated with and without the sensels on the edge of the contact region, and an average of the two was used as the contact area measurement. Differences between experimental and computational areas were recorded in both millimetres and percentage of the experimental area.

The computational contact force on each condyle was computed as the product of nodal contact area and nodal contact pressure, added across all nodes of the corresponding tibial condyle. The experimental equivalent was calculated from the Tekscan pressure grid over all sensels with non-zero pressure. The percentage of the total contact force experience by each condyle was then calculated and compared.

To provide context for those results, the movement of the tibia and the residual reaction forces and moments were extracted in the ‘partially freed’ computational cases to verify that the methodology used to free the degrees of freedoms one at a time generated the expected free motion (for which the residuals should be minimal).

## Results

The data associated with this paper: 3D images, experimental raw data, FE models, and processed results, are openly available from the University of Leeds Data Repository,^12^ as part of ‘the Institute of Medical and Biological Engineering Knee Dataset’.^23^

**Figure 3.**
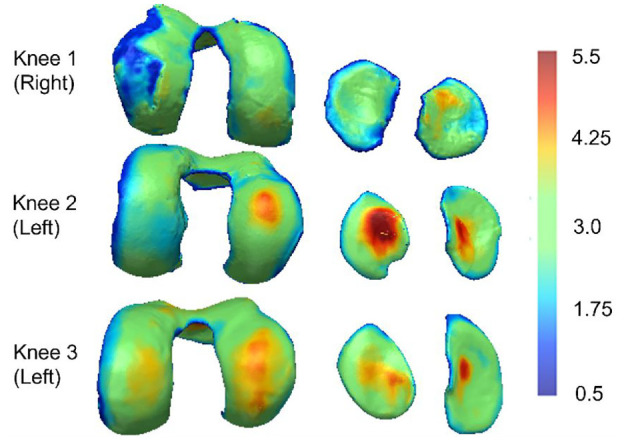
Colour maps describing the femoral and tibial cartilage thickness of the three knees (in mm). Thickness has been estimated using a sphere expansion algorithm and all plots are on the same colour scale. The cartilage thickness exceeded 5.5 mm in some regions.

The thickness distribution was different in the three knees (Figure 3). Knee 1 showed signs of cartilage degeneration, including irregular thickness distribution and extensive thin areas, particularly on the lateral condyle. Knees 2 and 3 both had thickness distributions more consistent with healthy tissue, but the peak thickness and distribution varied between those knees and condyles.

### Effect of cartilage thickness distribution, with constrained tibiofemoral alignment

**Figure 4.**
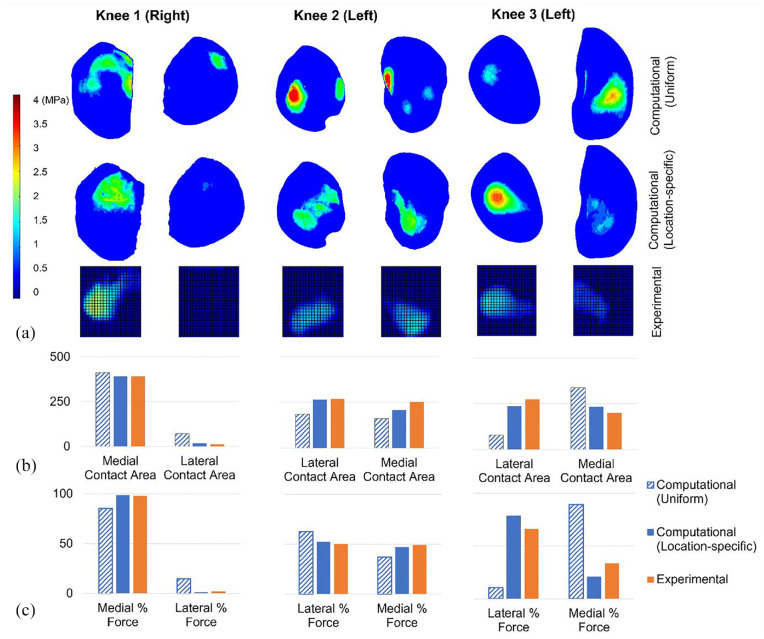
Computational-experimental comparison of tibiofemoral contact mechanics with a fixed relative alignment (fully constrained cases). (a) A visual comparison of the contact pressure distribution. Computational and experimental images are presented on the same spatial scale but the relative alignment is approximate. The contact pressure scale applies to all pressure visualisations. (b) The contact area (in mm^2^) on the medial and lateral tibial condyles. All graphs on each share the same contact area axis scale. (c) The proportion of the total force passing through the medial and lateral condyles. All graphs on each share the same force percentage axis scale. Uniform and location-specific cartilage thickness cases are presented for comparison. All cases have a 500 N load applied.

In the fully constrained cases, the contact pressure maps from models with location-specific cartilage thickness (‘location-specific’ cartilage models) were qualitatively more consistent with the experimental measurements than those from models with uniform thickness cartilage (‘uniform’ cartilage models; Figure 4(a)). Specifically, in Knees 1 and 2, the uniform cartilage models generated a more distributed and disjointed pressure map, in comparison to the location-specific cartilage models and the experimental pressure maps. For Knee 3, the use of a uniform cartilage layer substantially affected the distribution of pressure between the two condyles, generating a larger contact patch with more focused pressure on the medial side, in contrast to the experimental case. Similarly, the uniform case generated a contact patch on the lateral side of Knee 1, which was largely absent in both the experimental and location-specific computational cases.

The magnitude of the contact area on each tibial condyle was predicted more successfully by the location-specific cartilage models (absolute error range <1 mm^2^–43 mm^2^ or <1%–34%) compared to the uniform cartilage models (absolute error range 22 mm^2^–195 mm^2^ or 6%–315%). Raw area results are visualised in Figure 4(b). Overall the prediction of contact area was weakest for Knee 3.

The proportion of the force through each tibial condyle recorded experimentally was successfully replicated by the location-specific cartilage models for Knee 1 (<1% difference) and for Knee 2 (∼2% difference) but was less precise for Knee 3 (13% difference). Overall, the difference to the experimental measurement was lower in the location-specific cartilage models (from <1% to 13%) than in the uniform cartilage models (from 12% to 56%). For Knee 3, the use of a uniform cartilage layer generated a large error in the distribution of force between the two condyles (Figure 4(c)).

### Effect of cartilage thickness distribution, with partially freed tibiofemoral alignment

**Figure 5.**
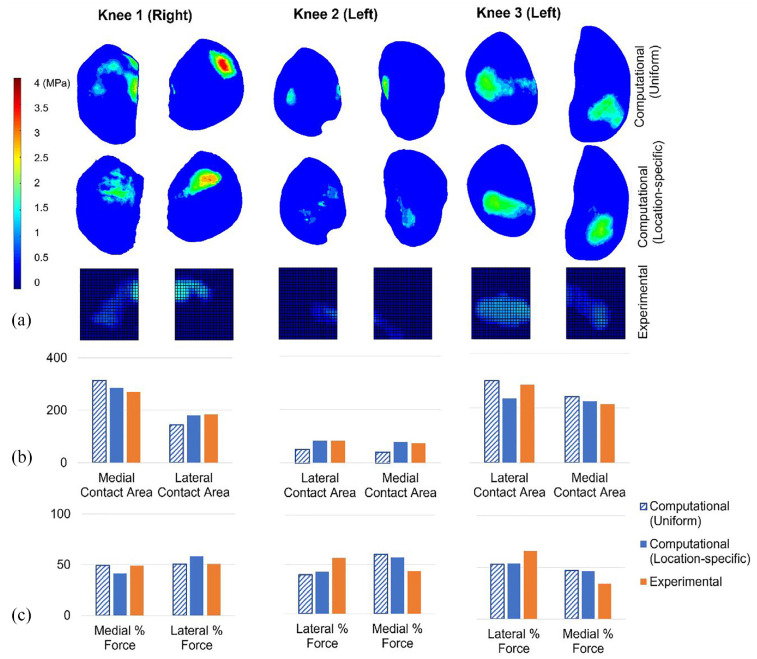
Computational-experimental comparison of tibiofemoral contact mechanics with freedom of relative alignment (partially freed cases). (a) A visual comparison of the contact pressure distribution. Computational and experimental images are presented on the same spatial scale but the relative alignment is approximate. The contact pressure scale applies to all pressure visualisations. (b) The contact area (in mm^2^) on the medial and lateral tibial condyles. All graphs on each share the same contact area axis scale. (c) The proportion of the total force passing through the medial and lateral condyles. All graphs on each share the same force percentage axis scale. Uniform and location-specific cartilage thickness cases are presented for comparison. Knees 1 and 3 have a 500 N load applied and Knee 2 has an 80 N load applied.

In cases with less constraint on relative bone alignment, there was some evidence that the ‘location-specific’ cartilage models qualitatively matched the patterns of experimental contact pressure better than the ‘uniform’ cartilage models (Figure 5(a)). For Knees 1 and 3 the ‘location-specific’ case predicted more consolidated contact patches, consistent with the experimental data, and for Knee 2 the location of the contact patch on the medial side was better predicted, as it was placed more posteriorly, consistent with the experiment.

The contact area on each tibial condyle was predicted more successfully by the location-specific cartilage models (absolute error range <1 mm^2^–51 mm^2^ or <1%–18%) compared to the uniform cartilage models (absolute error range 15 mm^2^–44 mm^2^ or 5%–45%). Contact areas for each condyle are visualised in Figure 5(b). Overall, both cartilage representations matched the experimental results better in the partially freed case compared to the fully fixed case (Table 1). The advantage of the location-specific approach, over the uniform approach, was reduced in the partially freed case. The experimental contact regions in the partially freed case often reached the inner edge of the sensor, meaning that those measurements may be slightly underestimated. The sensors were placed to sit against the intercondylar eminence (Figure 1) meaning that the underestimation should be minimal.

**Table 1.** Average error in the computational models when compared to the experimental data in terms of contact area and force balance. Mean values given across all six condyles.

Cartilage representation	Tibial constraint	Mean error in the percentage of force carried by each condyle (%)	Mean absolute error in contact area (mm^2^)
Location-specific	Fixed	5	21
Uniform thickness	Fixed	27	98
Location-specific	Freed	11	14
Uniform thickness	Freed	10	32

In the prediction of the proportion of force transferred through each condyle (Figure 5(c)) there was no advantage to the location-specific cartilage. The match to the experimental data was better for the uniform case for Knee 1, slightly better for the location-specific case for Knee 2 and similar in both cases for Knee 3. This measure appears to be sensitive to the final orientation of the tibia in both computational and experimental tests. The computational method used to replicate the case with three degrees of freedom freed on the tibia resulted in zero residual reaction moment in adduction–abduction, but there remained some reaction forces in the anterior–posterior direction and reaction moments in internal–external rotation, as these were prioritised lower in the methodology. (Reaction forces are detailed in Supplementary Material.) For Knees 1 and 2 there was a reduction in those reaction forces compared to the fully fixed scenario, and the predicted movements were small (adduction–abduction and internal–external rotations <1° and anterior–posterior translation <1 mm). For Knee 3 the anterior–posterior translation and internal–external rotation where larger (∼5 mm and ∼5° respectively). The larger movement in Knee 3 agreed with an experimental observation that Knee 3 generated an anterior tibia translation of approximately 10 mm. (The tibial movements were not systematically recorded experimentally.) There was a high reaction moment in the constrained flexion–extension, implying that a tibial slope was driving the larger tibial movements. In this case, while the reaction moments in internal–external rotation reduced compared to the fixed case, the reaction force in anterior–posterior direction increased.

## Discussion

### Benefit of location-specific cartilage thickness under constrained conditions

Under constrained conditions, where the effects of geometry are isolated, the benefit of using location-specific thickness can be quantified. The mean errors in force balance and contact area across all six condyles were substantially lower for the location-specific cartilage thickness, compared to the uniform cases, and the configuration of contact patches matched the experimental data better. These results support the use of location-specific cartilage thickness to replicate subject-specific experimental contact mechanics in the human tibiofemoral joint. This conclusion agrees with studies on the human elbow model,^24^ the murine knee^25^ and the human hip.^26^

Where truly subject-specific geometry is needed, there is therefore a need for image methodologies that enable a clear delineation of the articular surfaces and that have a resolution sufficient to achieve good cartilage thickness distribution representation.^24^ Whilst in the current in vitro study this could be achieved with computed tomography of the bone and cartilage layers after the knee dislocation (in common with other studies^24–26^) other imaging techniques, such as high-resolution MR imaging, may be more adapted for in vivo studies.^27–30^ In this work, the use of CT images of the separated tibial and femoral bones allowed for a consistent segmentation process, with little user-dependency, providing confidence that the thickness variation was well captured, provided it was larger than the image resolution. However, this image quality is not consistently available in vivo. While sub-millimetre resolution can be achieved on 3T MR imaging systems (e.g. 0.35 mm in plane^27^), out-of-plane resolution and distinction between tissues remain challenging.

Where subject-specific detail is not essential, the cartilage shape could however be extrapolated from atlas-based models^31^ or from image datasets at higher resolution,^23^ factoring in the variation in healthy cartilage thickness with knee shape.^32,33^ It is possible that Knees 2 and 3 (in this study) could have been generated using such a method. However, the cartilage wear apparent in Knee 1 would be more challenging to predict and require an extensive set of training images of knees with damaged cartilage.

This study is limited to three knees and therefore does not fully represent the possible cartilage thickness profiles but does include irregular variation due to damage (Knee 1) as well as smoother variations of healthier cartilage (Knees 2 and 3). The specimen-specific cartilage material properties were unknown and therefore estimated values were set consistently across all knees. This adds uncertainty to the contact mechanics predictions, but this is expected to be of a lower order than the effect of geometry. The load applied, while not at the higher end of physiological forces, was sufficient to generate substantial contact areas (and to induce tibial movement in the unconstrained cases). The good match between the location-specific cartilage model and the experimental data in the constrained case gave confidence that the experimental alignment was well replicated in the computational model, as small movements, e.g. <1º or 1 mm, in the unconstrained case caused large differences from the fixed case. The contact area and force comparisons were limited by the resolution of the experimental pressure sensors. Higher resolution measurements may show the benefit of location-specific cartilage more precisely but would not change the overall conclusion.

### Challenges of testing tibiofemoral joint with low constraint

With three degrees of tibial freedom, and therefore re-alignment of the joint during loading, the advantage of the location-specific cartilage thickness over uniform was not clear. The advantage of location-specific cartilage would be expected to persist when moving from the constrained to the unconstrained case, but it did not. There are several sources of uncertainty which may have caused this counterintuitive result including: the size and alignment of the pressure sensors, residual reaction forces, and a lack of stability under loading. In the partially freed case, reliably capturing the whole contact area on the pressure sensors was challenging. The centre of pressure was consistently captured however the contact area sometimes reached the edge of the sensor grid, and therefore some of the contact patch was not recorded. This affected the contact area measurements (and to a lesser degree, the proportion of force through each condyle). The removal of all constraint in three degrees of tibial freedom was challenging for the finite element solution process, where allowing three degrees of freedom simultaneously often caused the solution to fail. The approach devised to guarantee a solution resulted in some residual reaction forces and moments in the directions which were experimentally unconstrained. This indicates that the solution method did not reach exactly the result of a model with all three degrees of freedom unconstrained at the same time. The tibial movements were not recorded in the experimental tests and therefore it was unclear what the influence of friction and equipment limitations, such as bearings not being completely free floating, were in restricting the re-alignment of the joints. Since the computational and experimental motion uncertainties are from different sources, they each add to uncertainty and cannot be equated. The exposed cadaveric tibiofemoral joint, without ligaments, was vulnerable to instability, as illustrated by the lower level of force achieved for Knee 2.

### Contact mechanics sensitivity to cartilage thickness distribution under constrained conditions

The prediction of force proportion experienced by each condyle using uniform cartilage cases was substantially better under the partially freed scenario, than under the fixed alignment scenario (Table 1). While noting that free scenario involves a large amount of measurement uncertainty, a mechanical explanation can be offered for this difference. Where some regions of the uniform cartilage are thicker than the corresponding cadaveric tissue, they may be coming into contact early in the simulation process. Under constrained conditions these regions remain in contact and develop high pressure as the load increases, which generates different contact areas and load balance results to the cadaveric case. In the partially freed case, the tibia could move in reaction to this contact force, causing a redistribution of the areas of contact, particularly where there were larger imbalances between condyles in the fully constrained case (i.e. for Knees 1 and 3).

This idea is supported by work describing the mechanism by which contact pressures seen in their uniform cartilage cases were likely to be increased by the constraints imposed on the joint.^24^ In another study, it was necessary to generate different thicknesses of uniform layers on each knee condyle in order to separate the effect of alignment from the effect of thickness distribution, in the constrained conditions they applied.^25^

In many cases only low resolution or low contrast imaging may be available for model construction,^34,35^ making it necessary to use uniform, or otherwise approximated cartilage layer thickness. In those circumstances, it may be wise to avoid highly constrained test conditions and that allowing some freedom of movement may reduce the effects of cartilage geometry imprecision.

## Conclusions

The combined experimental and computational work in this study demonstrated the importance of cartilage geometry in finite element prediction of the contact mechanics of three human tibiofemoral joints, under constrained conditions. Compared to uniform cartilage thickness, subject-specific thickness profiles on the tibia and femur resulted in substantially better prediction of the experimental contact area (error of 21 mm^2^ vs 98 mm^2^) and force balance between condyles (error of 5% vs 27%).

The attempt to allow three degrees of relative freedom at the tibiofemoral joint, in the absence of ligamentous constraints, resulted in a high degree of experimental and computational uncertainty. It is therefore recommended that researchers avoid this level of freedom.

This work provides some evidence that highly constrained conditions make tibiofemoral contact mechanics predictions more sensitive to cartilage thickness and should perhaps be avoided in studies where the high-resolution, high-contrast images needed to generate subject-specific cartilage thickness are not available.

## Supplemental Material

sj-docx-1-pih-10.1177_09544119241307793 – Supplemental material for The role of high-resolution cartilage thickness distribution for contact mechanics predictions in the tibiofemoral joint Supplemental material, sj-docx-1-pih-10.1177_09544119241307793 for The role of high-resolution cartilage thickness distribution for contact mechanics predictions in the tibiofemoral joint by Robert J Cooper, Gavin A Day, Vithanage N Wijayathunga, Jiacheng Yao, Marlène Mengoni, Ruth K Wilcox and Alison C Jones in Proceedings of the Institution of Mechanical Engineers, Part H: Journal of Engineering in Medicine
