# Supplementary material for: The role of high-resolution cartilage thickness distribution for contact mechanics predictions in the tibiofemoral joint
Source: Proc Inst Mech Eng H. 2025 Jan 9;239(1):18–28. doi: 10.1177/09544119241307793 (PMC11894913; doi:10.1177/09544119241307793)
Supplement: sj-docx-1-pih-10.1177_09544119241307793 – Supplemental material for The role of high-resolution cartilage thickness distribution for contact mechanics predictions in the tibiofemoral joint [file sj-docx-1-pih-10.1177_09544119241307793.docx]

| **Case** | **Cart.** | **Const.** | **Knee** | **Contact Area (mm^2^)** | | **Difference exp-FE (mm^2^)** | | **Difference (%)** | | **Mean abs. diff. (mm^2^)** | **Mean abs. diff. (%)** |
| --- | --- | --- | --- | --- | --- | --- | --- | --- | --- | --- | --- |
|  |  |  |  | **Lateral** | **Medial** | **Lateral** | **Medial** | **Lateral** | **Medial** |  |  |
| **Exp.** |  | **Fixed** | **1** | **13.7** | **391.1** |  |  |  |  |  |  |
| FE | Seg. | Fixed | 1 | 18.3 | 391.9 | 4.6 | 0.8 | 33.6 | 0.2 |  |  |
| FE | Uni | Fixed | 1 | 71.5 | 413.2 | 57.7 | 22.1 | 315.3 | 5.7 |  |  |
| **Exp** |  | **Fixed** | **2** | **268.5** | **251.6** |  |  |  |  |  |  |
| FE | Seg | Fixed | 2 | 264.3 | 209.0 | -4.2 | -42.6 | -1.6 | -16.9 |  |  |
| FE | Uni | Fixed | 2 | 186.2 | 160.8 | -82.4 | -90.8 | -30.7 | -43.4 |  |  |
| **Exp** |  |  | **3** | **274.2** | **200.0** |  |  |  |  |  |  |
| FE | Seg | Fixed | 3 | 237.5 | 233.5 | -36.7 | 33.5 | -13.4 | 16.8 |  |  |
| FE | Uni | Fixed | 3 | 78.7 | 337.2 | -195.5 | 137.2 | -71.3 | 58.7 |  |  |
| FE | Seg | Fixed | ALL |  |  |  |  |  |  | 20.4 | 13.7 |
| FE | Uni | Fixed | ALL |  |  |  |  |  |  | 97.6 | 87.5 |
| **Case** | **Cart.** | **Const.** | **Knee** | **Contact Area (mm^2^)** | | **Difference exp-FE (mm^2^)** | | **Difference (%)** | | **Mean abs. diff. (mm^2^)** | **Mean abs. diff. (%)** |
|  |  |  |  | **Lateral** | **Medial** | **Lateral** | **Medial** | **Lateral** | **Medial** |  |  |
| **Exp.** |  | **Freed** | **1** | 185.5 | 270.2 |  |  |  |  |  |  |
| FE | Seg. | Freed | 1 | 181.2 | 286.1 | -4.3 | 15.9 | -2.3 | 5.9 |  |  |
| FE | Uni | Freed | 1 | 144.3 | 314.5 | -41.2 | 44.4 | -22.2 | 16.4 |  |  |
| **Exp** |  | **Freed** | **2** | 83.9 | 74.2 |  |  |  |  |  |  |
| FE | Seg | Freed | 2 | 83.8 | 79.9 | -0.1 | 5.7 | -0.1 | 7.6 |  |  |
| FE | Uni | Freed | 2 | 50.8 | 41.1 | -33.1 | -33.1 | -39.5 | -44.6 |  |  |
| **Exp** |  | **Freed** | **3** | 285.5 | 215.3 |  |  |  |  |  |  |
| FE | Seg | Freed | 3 | 234.7 | 225.0 | -50.8 | 9.7 | -17.8 | 4.5 |  |  |
| FE | Uni | Freed | 3 | 300.0 | 241.6 | 14.6 | 26.3 | 5.1 | 12.2 |  |  |
| FE | Seg | Freed | ALL |  |  |  |  |  |  | 13.4 | 6.4 |
| FE | Uni | Freed | ALL |  |  |  |  |  |  | 32.1 | 23.3 |

| **Case** | **Cart.** | **Const.** | **Knee** | **Load proportion through each condyle (%)** | | **Absolute difference exp-FE (%)** | **Mean abs. diff. (%)** |
| --- | --- | --- | --- | --- | --- | --- | --- |
|  |  |  |  | **Lateral** | **Medial** |  |  |
| **Exp.** |  | **Fixed** | **1** | 2 | 98 |  |  |
| FE | Seg. | Fixed | 1 | 1 | 99 | 1 |  |
| FE | Uni | Fixed | 1 | 15 | 85 | 13 |  |
| **Exp** |  | **Fixed** | **2** | 50 | 50 |  |  |
| FE | Seg | Fixed | 2 | 53 | 47 | 2 |  |
| FE | Uni | Fixed | 2 | 63 | 37 | 12 |  |
| **Exp** |  |  | **3** | 66 | 34 |  |  |
| FE | Seg | Fixed | 3 | 79 | 21 | 13 |  |
| FE | Uni | Fixed | 3 | 11 | 89 | 56 |  |
| FE | Seg | Fixed | ALL |  |  |  | 5 |
| FE | Uni | Fixed | ALL |  |  |  | 27 |
| **Case** | **Cart.** | **Const.** | **Knee** | **Load proportion through each condyle (%)** | |  | **Mean abs. diff. (%)** |
|  |  |  |  | **Lateral** | **Medial** |  |  |
| **Exp.** |  | **Freed** | **1** | **51** | **49** |  |  |
| FE | Seg. | Freed | 1 | 59 | 41 | 8 |  |
| FE | Uni | Freed | 1 | 51 | 49 | 0 |  |
| **Exp (80N)** |  | **Freed** | **2** | **57** | **43** |  |  |
| FE (80N) | Seg | Freed | 2 | 43 | 57 | 14 |  |
| FE (80N) | Uni | Freed | 2 | 40 | 60 | 17 |  |
| **Exp** |  | **Freed** | **3** | **66** | **34** |  |  |
| FE | Seg | Freed | 3 | 54 | 46 | 12 |  |
| FE | Uni | Freed | 3 | 53 | 47 | 13 |  |
| FE | Seg | Freed | ALL |  |  |  | 11 |
| FE | Uni | Freed | ALL |  |  |  | 10 |

| **Output type** | | | | **Tibial translation / rotations in degrees of freedom which are free in the “freed” cases** | | | **Tibial reaction forces / moments in degrees of freedom which are free in the “freed” cases** | | | **Tibial reaction forces / moments which are constrained in both “Fixed” and “Freed” cases** | | |
| --- | --- | --- | --- | --- | --- | --- | --- | --- | --- | --- | --- | --- |
| **Abaqus codes** | | | | **U2** | **UR2** | **UR3** | **RF2** | **RM2** | **RM3** | **RM1** | **RF1** | **RF3** |
| **Cart.** | **Const.** | **Knee** | **Load (N)** | **AP trans. (mm)** | **AA rot. (deg)** | **IE rot. (deg)** | **AP react. (N)** | **AA reaction moment - RM2 (Nmm)** | **IE reaction moment - RM3 (Nmm)** | **FE reaction moment - RM1 (Nmm)** | **ML reaction RF1 force (N)** | **SI reaction force RF3 (N)** |
| Seg | Fixed | 1 | 500 | 0 | 0 | 0 | -69 | 14400 | 1731 | -1283 | -112 | 500 |
| Seg | Freed | 1 | 500 | 0.71 | -1 | -0.83 | -19 | 0 | 45 | -2860 | -51 | 500 |
| Uni | Fixed | 1 | 500 | 0 | 0 | 0 | -59 | 10191 | 1212 | -1793 | -107 | 500 |
| Uni | Freed | 1 | 500 | 0.47 | -0.45 | -0.11 | -5 | 0 | 51 | -2412 | -94 | 500 |
| Seg | Fixed | 2 | 500 | 0 | 0 | 0 | -38 | 1754 | 856 | -245 | 48 | 500 |
| Seg | Freed | 2 | 500 | 0.4 | 0.09 | -0.93 | -30 | 0 | 416 | 21 | 56 | 500 |
| Seg | Freed | 2 | 80 | 0.13 | -0.19 | -0.58 | -7 | 0 | 81 | -64 | 6 | 80 |
| Uni | Fixed | 2 | 500 | 0 | 0 | 0 | -28 | 5964 | 380 | 626 | 119 | 500 |
| Uni | Freed | 2 | 500 | 0.07 | -0.36 | -0.15 | -4 | 0 | -20 | 1592 | 142 | 500 |
| Uni | Freed | 2 | 80 | -0.01 | -0.26 | 0.28 | -2 | 0 | -57 | 390 | 39 | 80 |
| Seg | Fixed | 3 | 500 | 0 | 0 | 0 | -48 | 6531 | 1450 | -6695 | 62 | 500 |
| Seg | Freed | 3 | 500 | 4.78 | -0.17 | -5.39 | -69 | 0 | 147 | -10409 | 7 | 500 |
| Uni | Fixed | 3 | 500 | 0 | 0 | 0 | -62 | -13058 | -393 | -7753 | 85 | 500 |
| Uni | Freed | 3 | 500 | 4.16 | 0.92 | -0.93 | -44 | 0 | 206 | -8867 | 23 | 500 |

**Abbreviations in tables:**

**Exp** = Experimental data

**FE** = finite element model data

**Seg** = segmented, location-specific cartilage thickness case

**Uni** = uniform cartilage thickness case

**Fixed** = all degrees of freedom constrained on the tibia

**Freed** = three degrees of freedom free on the tibia

**Tibial motions & reaction force notes**

These are the raw ones with L/R not resolved in the terms of direction.

Left: positive AA abduction; positive AP anterior; positive IE external

Right: positive AA adduction; positive AP anterior; positive IE internal
